# Non-contrast computed tomography at the end of the acute care phase as an additional prognostic tool after aneurysmal subarachnoid hemorrhage

**DOI:** 10.1007/s10143-026-04424-5

**Published:** 2026-08-04

**Authors:** Riccardo Ludovichetti, Massimo Barbagallo, Ignazio De Trizio, Mergime Maralushaj, Nathalie Nierobisch, Ramona-Alexandra Todea, Meritxell Garcia Alzamora, Zsolt Kulcsar, Giovanna Brandi

**Affiliations:** 1 Department of Neuroradiology, Clinical Neuroscience Center, University Hospital Zürich, Zürich, Switzerland; 2 Institute for Intensive Care Medicine, University Hospital Zurich and University of Zurich, Zurich, Switzerland; 3 Department of Neurology, Klinik Hirslanden Zurich, Zurich, Switzerland; 4 Diagnostic and Interventional Radiology, University Hospital Zürich, Zürich, Switzerland

**Keywords:** Aneurysmal subarachnoid hemorrhage, Computed tomography, Prognostication, Functional outcome

## Abstract

Aneurysmal subarachnoid hemorrhage (aSAH) is a life-threatening condition with high morbidity and mortality. Long-term outcomes remain difficult to predict. Computed tomography (CT) is central to diagnosis and early risk stratification, yet the prognostic value of non-contrast CT at the end of the acute care phase is not fully established. We conducted a retrospective single-center study of adult patients with confirmed aSAH admitted to the Neurocritical Care Unit (NCCU) of the University Hospital Zurich (January 2016–June 2024). Survivors with CT at discharge from the NCCU and 12-month follow-up were included. Functional outcome was assessed using the Glasgow Outcome Scale Extended (GOSE) and dichotomized as favorable (5–8) or unfavorable (1–4). CT scans were evaluated for infarcts, hemorrhage distribution, hydrocephalus requiring shunt, midline shift, and decompressive surgery. Multivariable logistic regression with multiple imputation by chained equations (MICE) addressed missing data, with days from admission to discharge CT included as a covariate in the clinical-severity model to control for differential timing. Discriminative performance was assessed using receiver operating characteristic (ROC) curve analysis. Of 500 admitted patients, 325 met inclusion criteria. At 12 months, 234 had favorable and 91 unfavorable outcomes. In a radiological model, increasing age and infarcts involving the thalamus, basal ganglia, corpus callosum, and cerebellum, together with cortical infarction and intraventricular hemorrhage, were independently associated with unfavorable outcome; sulcal subarachnoid hemorrhage showed only a non-significant trend toward a protective association. In a separate clinical-severity model, increasing age, ventriculoperitoneal shunt placement, decompressive craniectomy, and days-to-CT scan were independently associated with unfavorable outcome. ROC analysis showed low-to-moderate discriminative ability (AUC range 0.529–0.688), with intraventricular hemorrhage performing best. Discharge non-contrast CT provides incremental prognostic information in aSAH survivors. Deep or midline infarctions are particularly decisive for early prognostication and rehabilitation using routinely available imaging.

**Supplementary Information:**

The online version contains supplementary material available at 10.1007/s10143-026-04424-5.

## Introduction

Aneurysmal subarachnoid hemorrhage (aSAH), caused by the rupture of an intracranial aneurysm, is a life-threatening condition characterized by high morbidity and mortality [1]. Although many survivors experience progressive functional recovery, individual outcome trajectories remain difficult to predict [2, 3]. Imaging, particularly computed tomography (CT), plays a central role in diagnosis, follow-up, and risk stratification in this patient population [4]. 

CT is widely used in the acute setting owing to its rapid accessibility and effectiveness in identifying hemorrhage and predicting symptomatic vasospasm [5]. Previous studies have shown that factors such as hemorrhage burden, brain edema, and presence of intraventricular hemorrhage on acute-phase CT at admission correlate significantly with clinical outcomes [6, 7]. However, the prognostic significance of non-contrast CT acquired at the end of the acute care phase of aSAH has not yet been systematically explored. Although magnetic resonance imaging (MRI) offers higher sensitivity for detecting structural changes in the brain [8], its use is often limited by logistical constraints, availability, and patient instability in both the acute and post-acute settings [9]. Conversely, non-contrast CT remains a fast, robust, and widely available imaging modality that is routinely performed during the clinical course of aSAH. The final non-contrast CT obtained at the end of the acute care phase reflects the cumulative imaging status resulting from the entire hospitalization, integrating the effects of initial hemorrhage severity, secondary ischemic injury, hydrocephalus, and surgical or interventional treatments. While this imaging time point does not allow determination of the exact temporal onset of individual lesions, it provides a pragmatic summary of structural brain injury present at the transition from intensive care to recovery.

Continuous improvements in CT spatial resolution, supported by modern AI-based reconstruction techniques, enhance the detection of clinically relevant anatomical changes [10]. Interest in the prognostic value of non-contrast CT performed at the end of the acute care phase is further driven by the marked heterogeneity of long-term outcomes after aSAH, which range from complete recovery to severe, persistent disability [11, 12]. These outcomes are influenced by patient-related factors such as age and comorbidities, radiological findings and the clinical course during and after the acute care phase [13, 14]. Collectively, these considerations underscore the need to evaluate whether non-contrast CT performed at the end of the acute care phase could serve as an additional tool to guide prognostication, rehabilitation planning, and resource allocation in aSAH survivors. We specifically note that this analysis is designed as a complementary tool to established acute-phase prognostic scoring systems (WFNS, Hunt & Hess grades), not as a replacement.

The aim of this study is to systematically evaluate the prognostic value of non-contrast CT imaging acquired at the end of the acute care phase of aSAH and to investigate its association with one-year functional outcomes. By assessing specific radiological features present at discharge from the Neurocritical Care Unit, we sought to determine whether routine CT findings could provide reliable and accessible prognostic information regarding long-term functional recovery.

## Methods

### Study design and setting

This study received approval from the Cantonal Ethics Committee of Zurich (BASEC 2022 − 00270) and was conducted in accordance with the ethical principles established by the 1964 Declaration of Helsinki, including its subsequent amendments. Reporting of the study was structured in accordance with the Strengthening the Reporting of Observational Studies in Epidemiology (STROBE) guidelines [15]. 

### Participants, inclusion and exclusion criteria

All consecutive adult patients admitted to the Neurocritical Care Unit (NCCU) of the University Hospital of Zurich with confirmed aSAH between January 2016 and June 5, 2024, were considered eligible for this retrospective study. Inclusion criteria were (1) age ≥ 18 years and (2) confirmed diagnosis of aSAH, either by computed tomography or lumbar puncture. Exclusion criteria were: (1) in-hospital death during the intensive phase of care, (2) patients who explicitly refused, or whose legal representatives declined, to provide informed consent for the use of their medical data for research purposes, and (3) patients who underwent only magnetic resonance imaging (MRI). The analysis focuses on survivors of the acute care phase and therefore does not aim to predict early mortality.

### Clinical management

All patients were treated according to our institutional protocol which is based on the last available guidelines for management of patients with aSAH [16]. 

### Data collection and radiological assessment

Data collection was performed by reviewing the electronic clinical information system (KISIM-TM; Cistec, Zurich, Switzerland) for demographic and clinical information, and the local Picture Archiving and Communication System archive (PACS, DeepUnity Diagnost, Dedalus Healthcare, Germany) for radiological data. Demographic data included age and sex. Clinical information included the date of admission to the NCCU, date of discharge, duration of hospital stay, timing of non-contrast CT relative to admission (in days), and functional clinical outcome. The timing of discharge CT in our cohort varied based on clinical trajectory, and we address this potential source of confounding through statistical adjustment. Aneurysm treatment modality was categorized as either neurosurgical intervention (microsurgical clipping, bypass grafting, aneurysm trapping, or wrapping) or neuroradiological intervention (endovascular coiling or other catheter-based techniques). This two-category classification captures the fundamental distinction between open surgical and endovascular approaches to aneurysm repair while avoiding excessive subcategorization. Clinical outcome was assessed at 12 months after discharge from the NCCU using the Glasgow Outcome Scale Extended (GOSE) through a structured questionnaire [17, 18]. This evaluation included a neurological examination and a systematic assessment of the patient’s current occupational situation. The GOSE was dichotomized as unfavorable outcome (GOSE 1 to 4) and as favorable outcome (GOSE 5 to 8), as previously reported [19–21]. Radiological evaluation was based on CT scans obtained at the end of the acute care phase, following standardized institutional protocols. All scans were initially reviewed by the attending neuroradiologist at the time of scanning. For this study, a second neuroradiologist (RL) with seven years of experience in neurovascular imaging reviewed the scans to extract the relevant features for analysis. The following radiological features were systematically assessed as dichotomous variables (present/absent):

- Thalamic infarction: Presence of ischemic damage localized within the thalamic nuclei.
- Basal ganglia infarction: Presence of ischemic lesions affecting the caudate nucleus, putamen, globus pallidus, or related structures.
- Corpus callosum infarction: Presence of ischemic lesions involving the callosal fibers, including genu, body, and splenium.
- Brainstem infarction: Presence of lesions within the midbrain, pons, or medulla oblongata.
- Cerebellar infarction: Presence of ischemic lesions within the cerebellar hemispheres or vermis.
- Cortical infarction: Presence of ischemic lesions involving the cerebral cortex outside the deep gray matter structures.
- Sulcal subarachnoid hemorrhage: Presence of blood within the cortical sulci.
- Cisternal subarachnoid hemorrhage: Presence of hemorrhage within the basal cisterns, including the perimesencephalic, prepontine, and interpeduncular cisterns.
- Intraventricular hemorrhage (IVH): Presence of blood within the lateral, third, or fourth ventricles.
- Intracerebral hemorrhage: Presence of hemorrhage within the brain parenchyma, excluding blood confined to the subarachnoid space or ventricular system.
- Midline shift ≥ 3 mm: Presence of displacement of the brain midline structures from their normal anatomical position.
- Ventriculoperitoneal shunt: Presence of permanent ventriculoperitoneal shunts.
- Decompressive craniectomy: Evidence of decompressive craniectomy or bone flap removal.

**Fig. 1:**
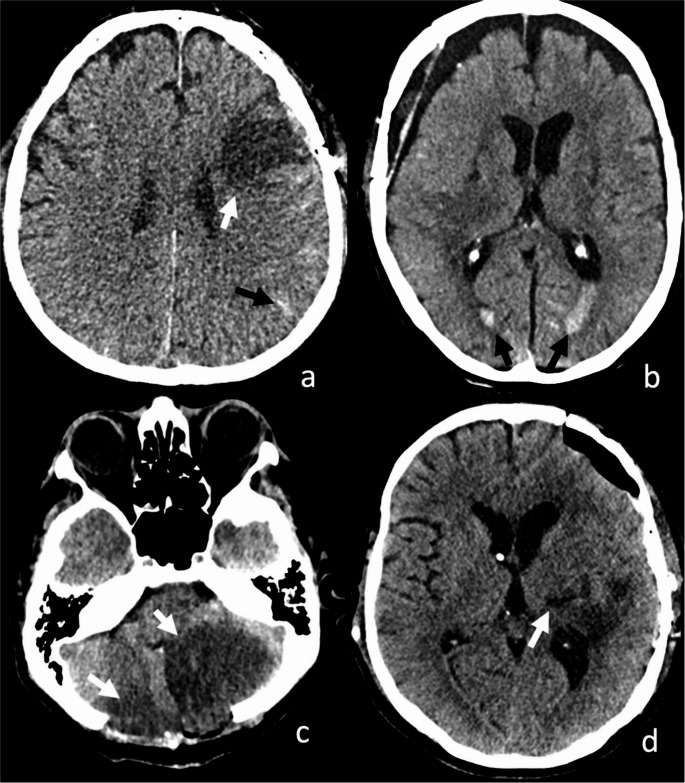
Examples of assessed dichotomous radiological variables. (**a**) Axial non-contrast CT showing a left frontal cortical infarct (white arrow) and subarachnoid hemorrhage along the left convexity (black arrow). (**b**) Axial CT demonstrating bilateral intraventricular hemorrhage within the occipital horns (black arrows). (**c**) Axial CT showing bilateral cerebellar infarcts (white arrows). (**d**) Axial CT demonstrating a left thalamic infarct (white arrow)

Infarcts identified on discharge CT were assessed as lesions of new onset compared to the earliest available non-contrast CT obtained at hospital admission, thereby differentiating acute sequelae of aSAH from pre-existing incidental findings. Because admission non-contrast CT in aSAH frequently contains thick subarachnoid blood, artifacts, or early edema that can obscure subtle baseline parenchymal lesions, we acknowledge that in a small number of cases a truly pre-existing infarct could not be entirely excluded with certainty; this is addressed further as a limitation below. Radiological features were recorded as dichotomous (present/absent) variables rather than by volume or extent, given that a continuous or graded measure would have created subgroups too small to analyze meaningfully in this cohort; however, an important qualitative dimension of infarct burden was retained through infarct location (e.g., thalamic, basal ganglia, corpus callosum, brainstem, cerebellar, or cortical), which was analyzed as a distinct variable and was independently associated with outcome. All radiological features were extracted from the local PACS archive in a structured manner to ensure consistency and accuracy. Examples of selected radiological features are shown in Fig. 1.

### Statistical analysis

Statistical analysis was performed using R version 4.5.1 [22]. Descriptive statistics are presented as counts and percentages for categorical variables, while medians with interquartile ranges are illustrated for continuous variables. Categorical variables were compared using Pearson’s χ² or Fisher’s exact test, whereas continuous or ordinal variables were analyzed with the Mann–Whitney U-test. Missing data were handled using multiple imputation by chained equations (MICE) [23]. Descriptive statistics (Table 1) were generated from the original dataset prior to imputation, while regression analyses were performed across all imputed datasets with pooling according to Rubin’s rules.

Days from hospital admission to discharge CT scan was included as a covariate in the clinical-severity multivariable model to account for potential confounding-by-indication due to differential timing of imaging between outcome groups. Multivariable analysis was performed using a generalized linear model (GLM) with a dichotomized GOSE at 12 months (unfavorable outcome versus favorable outcome). The estimates are reported as odds ratios (OR) for unfavorable outcome: an OR > 1 indicates an increased probability of an unfavorable outcome, an OR < 1 indicates a decreased probability of an unfavorable outcome, and an OR = 1 indicates no effect. To account for multiple testing within the multivariable logistic regression model, p-values were adjusted using the Bonferroni correction. A two-sided p-value < 0.05 after adjustment was considered statistically significant. The discriminatory performance of the model was evaluated by calculating the area under the receiver operating characteristic (ROC) curve with 95% confidence intervals. Values above 0.9 are considered of very strong prognostic value, 0.8–0.9 of good prognostic value, 0.7–0.8 of acceptable prognostic value, and < 0.7 of low prognostic value.

## Results

Of 500 patients admitted, 67 died during hospitalization (57 in the intensive care unit). Among the 433 survivors, 37 declined informed consent. Of the remaining 396 patients, 325 had available 12-month clinical follow-up and a non-contrast CT performed at the end of the acute care phase and were therefore included in the analysis. One patient with available 12-month clinical follow-up but MRI-only imaging during the late acute care phase was excluded. Demographic characteristics and radiological findings on non-contrast CT at the end of the acute care phase are summarized in Table 1.

**Table 1:** Baseline characteristics and radiological findings stratified by 12-month Glasgow Outcome Scale Extended (GOSE) outcome (favorable = GOSE 5–8; unfavorable = GOSE 1–4) in 325 aSAH patients. Data are median [IQR] or n (%). All radiological features classified as new lesions compared to admission imaging

Variable	Poor outcome (*n* = 91)	Favourable outcome (*n* = 234)	*p* value
Age, years, median [IQR]	59.00 [52.00, 71.00]	54.00 [47.00, 64.00]	0.001
Female sex, n (%)	59 (64.8)	150 (64.1)	1.000
Length of hospital stay, days, median [IQR]	25.00 [18.00, 33.00]	14.00 [12.00, 17.75]	< 0.001
Thalamic infarction, n (%)	14 (15.4)	3 (1.3)	< 0.001
Basal ganglia infarction, n (%)	24 (26.4)	12 (5.1)	< 0.001
Corpus callosum infarction, n (%)	32 (35.2)	17 (7.3)	< 0.001
Brainstem infarction, n (%)	7 (7.7)	3 (1.3)	0.008
Ventriculoperitoneal shunt, n (%)	44 (48.4)	45 (19.3)	< 0.001
Sulcal subarachnoid hemorrhage, n (%)	55 (60.4)	147 (62.8)	0.787
Cisternal subarachnoid hemorrhage, n (%)	11 (12.1)	29 (12.5)	1.000
Intraventricular hemorrhage, n (%)	72 (79.1)	94 (40.2)	< 0.001
Midline shift, n (%)	38 (41.8)	39 (16.7)	< 0.001
Decompressive craniectomy, n (%)	22 (24.2)	12 (5.1)	< 0.001
Cerebellar infarction, n (%)	5 (5.5)	3 (1.3)	0.072
Cortical infarction, n (%)	59 (64.8)	74 (31.6)	< 0.001
Intracerebral hemorrhage, n (%)	29 (31.9)	62 (26.6)	0.418
Days-to-CT scan, median [IQR]	22.00 [17.00, 31.00]	11.00 [5.00, 17.00]	< 0.001
Hunt & Hess Grade, n (%)			< 0.001
1	6 (6.6)	62 (26.6)	
2	12 (13.2)	75 (32.2)	
3	27 (29.7)	47 (20.2)	
4	23 (25.3)	26 (11.2)	
5	23 (25.3)	23 (9.9)	
WFNS Grade, n (%)			< 0.001
1	16 (17.6)	99 (42.5)	
2	12 (13.2)	58 (24.9)	
3	6 (6.6)	11 (4.7)	
4	28 (30.8)	37 (15.9)	
5	29 (31.9)	28 (12.0)	
Treatment = Neurosurgery, n (%)	43 (48.3)	103 (44.6)	0.635

*GOSE* Glasgow Outcome Scale Extended, *WFNS* World Federation of Neurological Surgeons grade; H&H, Hunt & Hess grade, *LOS* length of hospital stay, *IQR* interquartile range. Patients were categorized by treatment modality as either neurosurgical or neuroradiological intervention. Percentages are calculated among patients with available (non-missing) data for each variable; denominators therefore vary slightly across rows

Based on the 12-month GOSE assessment, 234 patients had a favorable outcome (GOSE 5–8) and 91 had an unfavorable outcome (GOSE 1–4). Patients with favorable outcomes were significantly younger (median 54 [47–64] vs. 59 [52–71] years, *p* < 0.001, independent of sex) and had shorter hospital stays in the NCCU (median 14 [12–17.75] vs. 25 [2–4, 6, 10, 12, 14, 21, 24–31] days, *p* < 0.001). On CT imaging, patients with favorable outcomes exhibited fewer structural abnormalities, particularly infarcts involving the thalamus, basal ganglia, or corpus callosum. They also had lower rates of ventriculoperitoneal shunt placement, intraventricular hemorrhage, and history of decompressive craniectomy (Table 1).

To disentangle the independent contribution of imaging findings from clinical severity markers that are not purely radiological in nature, multivariable analysis was performed using two separate models. The radiological model included imaging-derived variables together with age and sex (Table 2). The clinical-severity model included ventriculoperitoneal shunt placement, decompressive craniectomy, days-to-CT scan, admission severity grades (Hunt & Hess, WFNS), and treatment modality, together with age and sex (Table 3).

In the radiological model, increasing age and infarcts involving the thalamus, basal ganglia, corpus callosum, and cerebellum, together with cortical infarction and intraventricular hemorrhage, were independently associated with unfavorable 12-month outcome (Table 2). Brainstem infarction was not associated with outcome.

In the clinical-severity model, increasing age, ventriculoperitoneal shunt placement, decompressive craniectomy, and days-to-CT scan were independently associated with unfavorable 12-month outcome, as was Hunt & Hess grade 3 relative to grade 1 (Table 3). WFNS grade and treatment modality (neurosurgical vs. neuroradiological) were not independently associated with outcome in this model.

**Table 2:** Multivariable logistic regression model for the association of radiological findings with unfavorable 12-month outcome (GOSE 1–4) in aSAH. Model includes CT-derived radiological variables together with age and sex. OR, odds ratio; CI, confidence interval. Data analyzed with multiple imputation by chained equations (MICE) for missing variables

Variable	Odds ratio	95% CI	*p* value
Age (per year increase)	1.05	1.02–1.08	< 0.001
Female sex	0.88	0.45–1.74	0.708
Thalamic infarction	8.82	1.73–56.25	0.012
Basal ganglia infarction	4.30	1.52–12.50	0.006
Corpus callosum infarction	6.39	2.82–14.95	< 0.001
Sulcal subarachnoid hemorrhage	0.53	0.26–1.06	0.073
Intraventricular hemorrhage	5.92	2.96–12.53	< 0.001
Brainstem infarction	0.98	0.09–8.12	0.982
Cortical infarction	2.16	1.13–4.15	0.020
Cerebellar infarction	7.15	1.23–54.68	0.037

**Table 3:** Multivariable logistic regression model for the association of clinical severity markers with unfavorable 12-month outcome (GOSE 1–4) in aSAH. Model includes ventriculoperitoneal shunt, decompressive craniectomy, days-to-CT scan, admission severity grades (Hunt & Hess, WFNS), and treatment modality (neurosurgical vs. neuroradiological), together with age and sex. OR, odds ratio; CI, confidence interval. Data analyzed with multiple imputation by chained equations (MICE) for missing clinical variables

Variable	Odds Ratio	95% CI	*p* value
Age (per year increase)	1.05	1.02–1.08	< 0.001
Female sex	0.90	0.47–1.71	0.741
Ventriculoperitoneal shunt	3.30	1.71–6.46	< 0.001
Decompressive craniectomy	6.72	2.56–18.56	< 0.001
Days-to-CT scan	1.02	1.01–1.05	0.040
Hunt & Hess Grade (Reference: Grade 1)			
Grade 2	1.52	0.45–5.60	0.507
Grade 3	5.67	1.27–28.51	0.027
Grade 4	6.52	1.03–45.00	0.050
Grade 5	3.08	0.34–28.39	0.315
WFNS Grade (Reference: Grade 1)			
Grade 2	0.48	0.14–1.51	0.222
Grade 3	0.58	0.10–3.12	0.534
Grade 4	0.61	0.12–2.91	0.541
Grade 5	1.11	0.16–7.78	0.918
Treatment = Neurosurgery	1.15	0.58–2.25	0.688

Receiver operating characteristic (ROC) analysis indicated limited to low-moderate discriminative ability for individual CT-derived features (Table 4), with intraventricular hemorrhage demonstrating the highest performance (AUC = 0.688). Other infarct locations, age, ventriculoperitoneal shunt placement, and decompressive craniectomy showed AUC values ranging from 0.529 to 0.630.

**Table 4:** Receiver Operating Characteristic (ROC) Analysis for Association with Unfavorable Outcome. Receiver operating characteristic (ROC) analysis was performed to assess the discriminative ability of individual clinical and CT-derived variables for identifying unfavorable outcome (GOSE 1–4). Observed AUC values indicate limited to moderate discriminative ability, with no single variable demonstrating strong standalone prognostic performance

Variable	AUC
Age	0.616
Thalamic infarction	0.572
Basal ganglia infarction	0.604
Corpus callosum infarction	0.630
Ventriculoperitoneal shunt	0.630
Intraventricular hemorrhage	0.688
Decompressive craniectomy	0.590
Cerebellar infarction	0.529

## Discussion

In this study, we examined whether the CT scan performed at discharge in survivors of the acute care phase of aSAH carries prognostic value for recovery at 12 months. The main observation is that certain findings on this routine scan—particularly deep infarctions involving thalamus, basal ganglia and corpus callosum—are consistently associated with poor functional outcome in the long term. These lesions may therefore constitute reliable imaging biomarkers that complement acute-phase severity scoring systems by capturing structural brain injury sustained during the NCCU stay.

These observations are particularly relevant in the context of the evolving clinical landscape of aSAH. While the condition still carries high early mortality, several recent population-level studies have shown that outcomes may improve over follow-up [24, 25, 29, 32]. As more patients survive the acute care phase, clinicians are increasingly confronted with individuals who fall into a “moderate severity” group—patients who are not catastrophically injured, but whose mid- and long-term outcome remains difficult to predict [7]. Uncertainty in these situations affects medical decision-making, family expectations, long-term care planning, and the allocation of rehabilitation resources. Tools that can help identify which patients may still improve are therefore highly valuable.

Non-contrast CT is universally available, rapid, and inexpensive, making it the standard imaging modality of choice for nearly all patients before discharge. This “snapshot” captures the cumulative effects of early brain injury, delayed ischemia, surgical or endovascular interventions, and hydrocephalus, allowing for practical prognostic assessment. MRI is well established as a prognostic tool in other acute brain injuries [28], but its use in aSAH is often limited by invasive monitoring devices, agitation, cardio-respiratory instability, and variable availability across centers [26]. The strong prognostic relevance of deep infarctions observed in this study is biologically plausible and consistent with prior literature [27, 31]. Infarcts involving the thalamus, basal ganglia and corpus callosum remained independently associated with unfavorable outcome after multivariable adjustment, underscoring the vulnerability of deep and midline structures. Injury to these regions disrupts circuits critical for arousal, attention, motor control, and cortico-subcortical network integration, which are essential for functional independence [27, 31]. This temporal difference likely reflects clinical trajectory rather than a systematic protocol deviation. Days-to-CT was included as a covariate in the clinical-severity model (Table 3), where markers of clinical severity remained independently associated with outcome after this adjustment; the radiological model was not itself adjusted for imaging timing, and this is addressed as a limitation below. Notably, basal ganglia infarction emerged as strongly and independently associated with poor long-term outcome, increasing the odds of unfavorable recovery about fourfold. Even small lesions in these structures may therefore carry a disproportionate burden of long-term disability, consistent with observations from traumatic brain injury studies [33, 34]. That these infarcts were clearly identifiable on routine discharge CT and remained significantly associated with functional outcome at 12 months underscores that non-contrast CT can provide clinically meaningful prognostic information, even for deep and midline injuries that are traditionally better characterized with MRI. Cortical and cerebellar infarction were also independently associated with unfavorable outcome in the radiological model, suggesting that the prognostic relevance of discharge CT extends beyond deep and midline structures to a broader pattern of structural brain injury. Other factors associated with unfavorable outcome included intraventricular hemorrhage, VP shunt placement, and decompressive craniectomy. Although these interventions reflect surgical management of acute complications, they serve as strong markers of initial hemorrhage severity. Unlike residual subarachnoid blood, which undergoes progressive reabsorption during recovery, the structural changes associated with VP shunt (altered cerebrospinal fluid physiology) and decompressive craniectomy (permanent loss of cranial vault protection) persist throughout recovery and independently influence long-term functional potential. Their prognostic significance thus reflects the cumulative burden of acute injury rather than a simple post-hoc marker of prior intervention. An important finding of this study is the limited relevance of residual subarachnoid blood on discharge CT to long-term outcome. Neither sulcal nor cisternal subarachnoid hemorrhage differed significantly between outcome groups on univariate comparison (Table 1), and sulcal hemorrhage showed only a non-significant trend toward a protective association in the multivariable radiological model (Table 2); cisternal hemorrhage was not carried forward into the multivariable model given its lack of univariate association.

While blood burden is highly relevant in the first days after hemorrhage due to its association with cerebral vasospasm and delayed cerebral ischemia [5, 35, 36], our data suggest that, beyond the acute period, residual subarachnoid blood does not independently contribute to long-term functional outcome. This distinction is important in everyday clinical practice, as early prognostic discussions often place considerable emphasis on hemorrhage burden and Fisher or (m)Fisher grading to establish a presumed severity of outcome [37], even though this does not necessarily reliably predict long-term functional outcome once the high-risk phase has passed.

A reasonable interpretation is that patients who make it through the early, unstable phase have already passed the period when blood breakdown products actively trigger complications such as vasospasm, cerebrospinal fluid (CSF) flow disturbance, or acute inflammatory responses [30]. After this time window, the remaining blood on imaging often reflects the original hemorrhage rather than a process that continues to shape neurological recovery.

Taken together, these results support the use of discharge non-contrast CT as a practical and readily available tool for long-term prognostication. Identification of deep or midline infarctions early can help refine expectations, guide rehabilitation planning, and facilitate clearer communication with patients and families. The low relevance of residual subarachnoid blood further emphasizes the importance of structural brain injury as a long-term determinant of functional outcome. Importantly, this approach is designed to provide additional prognostic information at the transition from intensive care—a clinically distinct time point not captured by acute-phase scoring systems (WFNS, Hunt & Hess grades)—and should not be construed as a replacement for established clinical predictors at admission.

It is important to define the population to which these findings apply. Patients who died during the acute care phase were excluded from this analysis, in part because this also removes deaths related to redirection of care that are not meaningfully explained by imaging findings; patients without 12-month follow-up were likewise excluded. Our model therefore does not characterize risk for the entire aSAH population, including those who do not survive the acute phase, but specifically for patients who survive to the point of discharge or late acute-phase imaging. Within this survivor population, we intend these findings as an additional, imaging-based tool to be used alongside existing clinical knowledge to support prognostic discussions with patients and their families, rather than as a population-wide risk-stratification instrument or a replacement for early mortality-risk assessment.

The study has several strengths. The analyses were performed on a well-characterized cohort, with missing data addressed using multiple imputation, and imaging was interpreted by two neuroradiologists, reducing the risk of classification bias. Importantly, the proposed markers are based on non-contrast CT, an examination that is available in virtually all hospitals and unaffected by renal failure, implanted hardware, or logistical constraints, enhancing generalizability across care settings.

Limitations must also be acknowledged. This is a retrospective, single-center study, which may limit generalizability. Discharge CT timing differed between outcome groups (median 11 vs. 22 days); we anchored the reference timepoint to NCCU discharge—a clinically meaningful endpoint—rather than a fixed postadmission day, and included days-to-CT as a covariate in the clinical-severity model, where markers of severity remained significant after adjustment. The radiological model itself was not adjusted for timing, so residual confounding-by-indication cannot be fully excluded for the infarct associations specifically. Infarcts were assessed as dichotomous rather than volumetric variables given sample-size constraints, and rare pre-existing lesions obscured by admission-CT artifact cannot be fully excluded; infarct location was retained as a qualitative proxy for lesion burden. Treatment modality was limited to two broad categories, pooling endovascular techniques with potentially different complication profiles. The 8-point GOSE was dichotomized rather than analyzed as an ordinal shift, since distributing 91 unfavorable-outcome events across 8 categories would have produced sparse-data bias. Our two multivariable models retain 10 and 14 parameters, respectively (EPV 9.1 and 6.5)—below the traditional EPV-10 threshold but within the permissive EPV 5–9 range supported by the methodological literature—and were not internally validated, as this would have required withholding roughly 30% of an already limited sample; reported performance should therefore be interpreted as apparent (in-sample) rather than externally validated. Finally, GOSE does not capture cognitive or emotional outcomes, and follow-up was limited to 12 months, although patients who continue to improve beyond this point are typically those who already demonstrated more favorable outcomes at 12 months [24, 38, 39]. Future multicenter studies with external validation are needed to confirm and refine these findings.

In conclusion, non-contrast CT performed at the end of the acute care phase provides incremental prognostic information in patients who survive aSAH. Deep or midline infarctions, when present, are consistently associated with unfavorable 12-month outcome and can help clinicians frame early discussions about recovery potential and rehabilitation planning. These findings derive from a retrospective, single-center cohort and warrant external validation; markers of clinical severity, meanwhile, remained independently associated with outcome even after adjusting for imaging timing, supporting the overall robustness of the discharge-CT approach. Rather than replacing acute-phase severity scores such as WFNS or Hunt & Hess, discharge CT offers complementary information at a distinct clinical transition, using imaging already obtained in routine practice — making it a practical, readily available addition to prognostic counseling at a critical point in patient care.

## Supplementary Information

Below is the link to the electronic supplementary material.

Supplementary Material 1

## Data Availability

The data supporting the findings of this study are not publicly available due to ethical and legal restrictions, as they contain information that could compromise participant privacy. Deidentified participant data are available from the corresponding author, Riccardo Ludovichetti (riccardo.ludovichetti@usz.ch), upon reasonable request and subject to approval by the relevant Ethics Committee. Data are provided for information purposes only and may not be reused.
