# Supplementary material for: Non-contrast computed tomography at the end of the acute care phase as an additional prognostic tool after aneurysmal subarachnoid hemorrhage
Source: Neurosurg Rev. 2026 Aug 4;49(1):510. doi: 10.1007/s10143-026-04424-5 (PMC13437646; doi:10.1007/s10143-026-04424-5)
Supplement: Supplementary file 1 [file 10143_2026_4424_MOESM1_ESM.docx]

SUPPLEMENTARY MATERIAL

**Supplementary Table 1. Baseline characteristics and radiological findings stratified by 12-month GOSE outcome in all 395 aSAH patients with multiple imputation by chained equations (MICE) for missing clinical variables.** Data are median [IQR] or n (%). Favorable outcome = GOSE 5–8; Unfavorable outcome = GOSE 1–4. MICE addresses missing data for WFNS and Hunt & Hess grades only; outcome variable remains as observed. Abbreviations: GOSE, Glasgow Outcome Scale Extended; WFNS, World Federation of Neurological Surgeons; H&H, Hunt & Hess; IQR, interquartile range.

| **Variable** | **Overall (n = 395)** | **Poor outcome (n = 106)** | **Favourable outcome (n = 289)** | **p value** |
| --- | --- | --- | --- | --- |
| **Age, years, median [IQR]** | 56.00 [47.00, 66.50] | 59.00 [52.00, 71.00] | 55.00 [46.00, 65.00] | 0.001 |
| **Female sex, n (%)** | 251 (63.5) | 69 (65.1) | 182 (63.0) | 0.787 |
| **Length of hospital stay, days, median [IQR]** | 15.00 [12.00, 22.00] | 24.50 [18.00, 33.00] | 14.00 [11.00, 17.00] | <0.001 |
| **GOSE Score, n (%)** |  |  |  | <0.001 |
| **1** | 26 (6.6) | 26 (24.5) | 0 (0.0) |  |
| **2** | 7 (1.8) | 7 (6.6) | 0 (0.0) |  |
| **3** | 28 (7.1) | 28 (26.4) | 0 (0.0) |  |
| **4** | 30 (7.6) | 30 (28.3) | 0 (0.0) |  |
| **5** | 40 (10.1) | 0 (0.0) | 40 (13.8) |  |
| **6** | 46 (11.6) | 0 (0.0) | 46 (15.9) |  |
| **7** | 58 (14.7) | 0 (0.0) | 58 (20.1) |  |
| **8** | 90 (22.8) | 0 (0.0) | 90 (31.1) |  |
| **NA** | 70 (17.7) | 15 (14.2) | 55 (19.0) |  |
| **GOSE dichotomized (Favourable outcome), n (%)** | 289 (73.2) | 0 (0.0) | 289 (100.0) | <0.001 |
| **Thalamic infarction, n (%)** | 21 (5.3) | 17 (16.0) | 4 (1.4) | <0.001 |
| **Basal ganglia infarction, n (%)** | 43 (10.9) | 29 (27.4) | 14 (4.8) | <0.001 |
| **Corpus callosum infarction, n (%)** | 56 (14.2) | 35 (33.0) | 21 (7.3) | <0.001 |
| **Brainstem infarction, n (%)** | 12 (3.0) | 8 (7.5) | 4 (1.4) | 0.005 |
| **Ventriculoperitoneal shunt, n (%)** | 105 (26.6) | 51 (48.1) | 54 (18.7) | <0.001 |
| **Sulcal subarachnoid hemorrhage, n (%)** | 245 (62.0) | 65 (61.3) | 180 (62.3) | 0.954 |
| **Cisternal subarachnoid hemorrhage, n (%)** | 48 (12.2) | 13 (12.3) | 35 (12.1) | 1.000 |
| **Intraventricular hemorrhage, n (%)** | 194 (49.1) | 81 (76.4) | 113 (39.1) | <0.001 |
| **Midline shift, n (%)** | 85 (21.5) | 41 (38.7) | 44 (15.2) | <0.001 |
| **Decompressive craniectomy, n (%)** | 39 (9.9) | 26 (24.5) | 13 (4.5) | <0.001 |
| **Intracerebral hemorrhage, n (%)** | 108 (27.3) | 32 (30.2) | 76 (26.3) | 0.521 |
| **Days to CT scan, median [IQR]** | 14.00 [6.00, 20.00] | 21.00 [15.25, 30.75] | 11.00 [4.00, 17.00] | <0.001 |
| **WFNS Grade, n (%)** |  |  |  | <0.001 |
| **1** | 144 (36.5) | 20 (18.9) | 124 (42.9) |  |
| **2** | 90 (22.8) | 13 (12.3) | 77 (26.6) |  |
| **3** | 19 (4.8) | 7 (6.6) | 12 (4.2) |  |
| **4** | 75 (19.0) | 34 (32.1) | 41 (14.2) |  |
| **5** | 67 (17.0) | 32 (30.2) | 35 (12.1) |  |
| **Hunt & Hess Grade, n (%)** |  |  |  | <0.001 |
| **1** | 80 (20.3) | 8 (7.5) | 72 (24.9) |  |
| **2** | 118 (29.9) | 15 (14.2) | 103 (35.6) |  |
| **3** | 84 (21.3) | 27 (25.5) | 57 (19.7) |  |
| **4** | 60 (15.2) | 30 (28.3) | 30 (10.4) |  |
| **5** | 53 (13.4) | 26 (24.5) | 27 (9.3) |  |
| **Treatment = Neurosurgery, n (%)** | 182 (46.1) | 49 (46.2) | 133 (46.0) | 1.000 |

**Supplementary Table 2. Receiver Operating Characteristic (ROC) analysis predicting unfavorable 12-month outcome including imaging features and treatment modality, excluding baseline clinical variables (age, WFNS, Hunt & Hess).** Demonstrates robustness of imaging findings independent of clinical scoring systems. AUC, area under the curve; CI, confidence interval. Data analyzed with MICE for missing variables.

| **Variable** | **AUC** | **95% CI** |
| --- | --- | --- |
| **Basal ganglia infarction** | 0.605 | 0.59–0.62 |
| **Corpus callosum infarction** | 0.626 | 0.61–0.64 |
| **Ventriculoperitoneal shunt** | 0.631 | 0.62–0.65 |
| **Intraventricular hemorrhage** | 0.686 | 0.67–0.70 |
| **Decompressive craniectomy** | 0.592 | 0.58–0.60 |
| **Cerebellar infarction** | 0.528 | 0.52–0.54 |

**Supplementary Table 3. Receiver Operating Characteristic (ROC) analysis predicting unfavorable 12-month outcome including imaging features and baseline clinical data, excluding treatment modality.** Demonstrates that imaging contributions remain significant independent of surgical approach. AUC, area under the curve; CI, confidence interval. Data analyzed with MICE for missing variables.

| **Variable** | **AUC** | **95% CI** |
| --- | --- | --- |
| **Age** | 0.619 | 0.60–0.64 |
| **WFNS Grade** | 0.685 | 0.67–0.70 |
| **Hunt & Hess Grade** | 0.708 | 0.69–0.72 |

**Supplementary Table 4. Receiver operating characteristic (ROC) curve analysis of individual variables predicting unfavorable 12-month outcome. AUC values (area under the curve) presented with 95% confidence intervals, derived from logistic regression models with multiple imputation (MICE).** Variables included: Age, WFNS grade, Hunt & Hess grade, thalamic infarction, basal ganglia infarction, corpus callosum infarction, ventriculoperitoneal shunt, intraventricular hemorrhage, decompressive craniectomy, cerebellar infarction, and days to CT scan. AUC, area under the curve; CI, confidence interval.

| **Variable** | **AUC** | **95% CI** |
| --- | --- | --- |
| **Age** | 0.619 | 0.60–0.64 |
| **Basal ganglia infarction** | 0.605 | 0.59–0.62 |
| **Corpus callosum infarction** | 0.626 | 0.61–0.64 |
| **Ventriculoperitoneal shunt** | 0.631 | 0.62–0.65 |
| **Intraventricular hemorrhage** | 0.686 | 0.67–0.70 |
| **Decompressive craniectomy** | 0.592 | 0.58–0.60 |
| **Cerebellar infarction** | 0.528 | 0.52–0.54 |
| **WFNS Grade** | 0.685 | 0.67–0.70 |
| **Hunt & Hess Grade** | 0.708 | 0.69–0.72 |
